# Haemorrhoidal bleeding as a co-manifestation of idiopathic pulmonary artery hypertension

**DOI:** 10.1093/ehjcr/ytae021

**Published:** 2024-01-08

**Authors:** Takao Konishi, Akimi Uehata

**Affiliations:** Department of Cardiovascular Medicine, Faculty of Medicine and Graduate School of Medicine, Hokkaido University, West 7, North 15, Kita-ku, Sapporo 060-8638, Japan; Division of Cardiology, Kisen Hospital, Tokyo, Japan

**Keywords:** Idiopathic pulmonary artery hypertension, Haemorrhoidal bleeding

The common symptoms of idiopathic pulmonary artery hypertension (IPAH) are breathlessness, exercise intolerance, leg oedema, and anorexia. We report a rare case of IPAH presenting with haemorrhoidal bleeding.

## Case description

**Figure 1:**
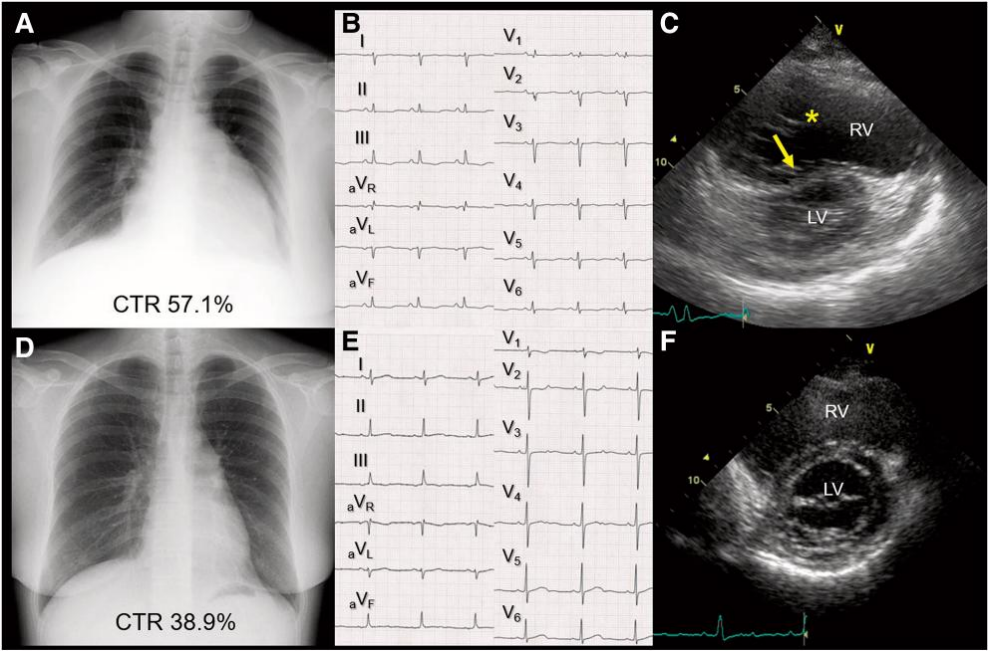
(*A*) Chest X-ray on admission. (*B*) An electrocardiogram on admission. (*C***)** A transthoracic echocardiogram on admission showing the dilatation of the right ventricle (asterisk) and the shift of intraventricular septum towards the left ventricle (arrow) (parasternal short-axis view). (*D*) Chest X-ray 2 months after admission. (*E*) An electrocardiogram 2 months after admission. (*F*) A transthoracic echocardiogram 2 months after admission (parasternal short-axis view). CTR, cardiothoracic ratio; LV, left ventricle; RV, right ventricle.

A 48-year-old female with a history of depression was admitted to our Department of Gastroenterological Surgery with acute haemorrhoidal bleeding. Prior to admission, she experienced pain during defecation and mild shortness of breath. Three days after admission, the patient was transferred to the cardiology department because of worsening dyspnoea. Physical examination revealed ascites and bilateral pitting oedema. Chest radiography revealed cardiac enlargement and bilateral pleural effusion (*Figure 1A*). Electrocardiography revealed a tall R wave in lead V_1_, a right-axis deviation of 117°, and deep S waves in the lateral leads, suggesting right ventricular hypertrophy (*Figure 1B*). Transthoracic echocardiography revealed prominent dilatation of the right ventricle (*Figure 1C*, asterisk) and a shift of the intraventricular septum towards the left ventricle (*Figure 1C*, arrow). Right heart catheterization revealed an elevated mean pulmonary artery pressure of 53 mmHg, a pulmonary artery wedge pressure of 12 mmHg, and an elevated pulmonary vascular resistance of 1454 dyn s/cm^5^. Additional multimodal examinations, including abdominal ultrasound, contrast-enhanced computed tomography, spirography, and pulmonary perfusion scintigraphy, excluded other aetiologies of pulmonary hypertension, confirming the diagnosis of IPAH. After the initiation of the vasodilator therapy, including beraprost, sildenafil, and bosentan, her haemorrhoids and cardiac symptoms improved. Chest radiography, electrocardiography, and echocardiography performed 2 months after admission were almost normal (*Figure 1D–F*).

## Discussion

Several studies have reported an association between haemorrhoidal disease and comorbidities such as obesity, cirrhosis, chronic obstructive pulmonary disease, and chronic thyroiditis,^1,2^ conditions that were ruled out in this case. The increased intra-abdominal pressure due to dyspnoea and the increased venous pressure due to IPAH may have led to the development of haemorrhoids. Further, the patient’s IPAH-related leg oedema and resulting restriction of water intake may have caused constipation,^3^ leading to the exacerbation of haemorrhoidal bleeding. Clinicians should recognize haemorrhoidal bleeding as a rare co-manifestation of worsening IPH-related dyspnoea, with the risk that delayed diagnosis can lead to a poor outcome.

## Data Availability

The data included in this article will be shared upon reasonable request to the corresponding author.

## Notes

**Consent:** The authors confirm that written consent for publication of this case report was obtained from the patient in accordance with COPE guidelines.

**Funding:** This work was supported in part by The Ito Foundation (The 27th Ito Foundation Grant, TK).
